# Atrial Cardiomyopathy: An Emerging Cause of the Embolic Stroke of Undetermined Source

**DOI:** 10.3389/fcvm.2021.674612

**Published:** 2021-08-09

**Authors:** Yuye Ning, Gary Tse, Guogang Luo, Guoliang Li

**Affiliations:** ^1^Stroke Centre and Department of Neurology, The First Affiliated Hospital of Xi'an Jiaotong University, Xi'an, China; ^2^Tianjin Key Laboratory of Ionic-Molecular Function of Cardiovascular Disease, Department of Cardiology, Tianjin Institute of Cardiology, Second Hospital of Tianjin Medical University, Tianjin, China; ^3^Kent and Medway Medical School, Canterbury, United Kingdom; ^4^Atrial Fibrillation Centre and Department of Cardiovascular Medicine, The First Affiliated Hospital of Xi'an Jiaotong University, Xi'an, China

**Keywords:** cardiac rhythm abnormalities, risk stratification, atrial cardiomyopathy, structural heart disease, embolic stroke of undetermined source

## Abstract

Nearly 30% of ischemic strokes have an unknown cause, which are referred to as cryptogenic strokes (CS). Imaging studies suggest that a large proportion of these patients show features that are consistent with embolism, and thus the term embolic stroke of undetermined source (ESUS) was proposed to describe these CS patients. Atrial cardiomyopathy predisposes to thrombus formation and thus embolic stroke even in the absence of atrial fibrillation (AF). This may provide a mechanistic link with ESUS, suggesting that anticoagulant therapy may be more beneficial than antiplatelet therapy in ESUS patients with atrial cardiomyopathy. The present review discusses the concept of atrial cardiomyopathy and ESUS and the relationship between them based on the mechanisms and clinical evidence, suggests that atrial cardiomyopathy may be a potential mechanism of ESUS, and highlights a theoretical basis that supports that anticoagulant therapy may be more applicable to ESUS patients with atrial cardiomyopathy and aims to help us better understand and identify the risk of ESUS, thereby improving the management of these patients in clinical practice.

## Introduction

**Figure 1:**
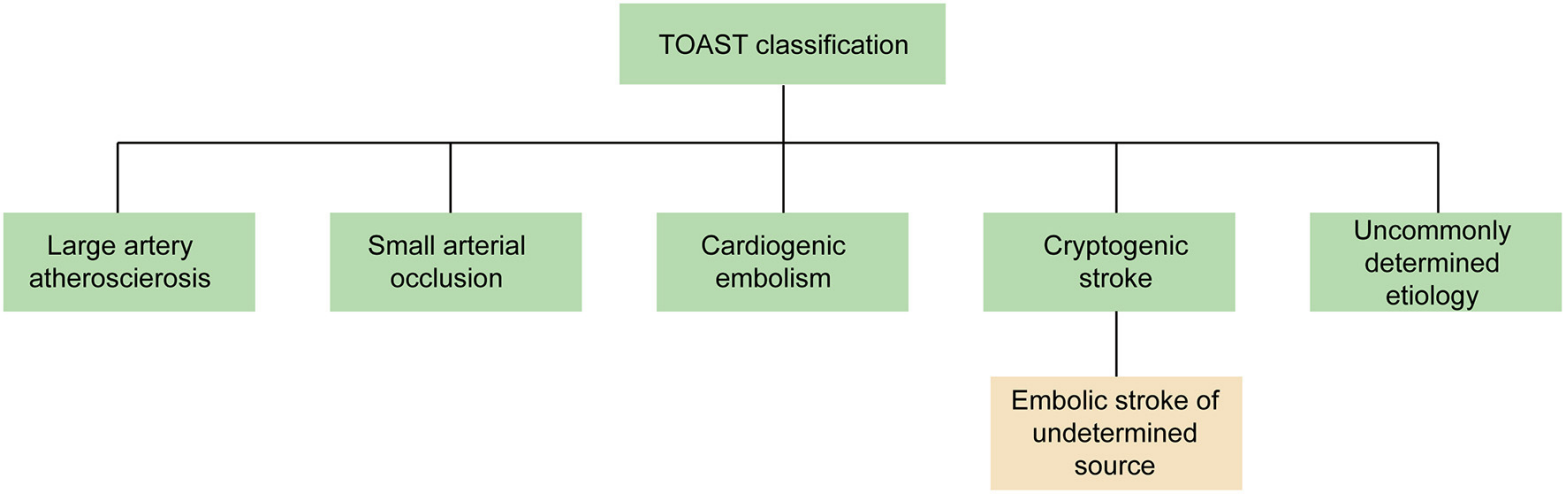
The classification of IS in TOAST. The term “ESUS” is a more specific and clinically useful concept to describe most of CS patients. TOAST, Trial of ORG 10172 in Acute Stroke Treatment; ESUS, embolic stroke of undetermined source; CS, cryptogenic stroke.

Several causes can lead to ischemic stroke (IS), including extracranial and intracranial large artery atherosclerosis, cardiogenic embolism, small arterial occlusion, and other uncommonly determined etiology, as detailed in the Trial of ORG 10172 in Acute Stroke Treatment (TOAST) (1). However, 30% of IS have no identifiable causes, which are referred to as cryptogenic stroke (CS). Imaging findings of CS patients demonstrated that up to 60% of CS patients had cortical infarction (2), which would suggest possible embolic origins. Therefore, the term “embolic stroke of undetermined source” (ESUS) was proposed in 2014 to describe non-lacunar IS patients without an identifiable cardioembolic source (Figure 1) (3).

Atrial cardiomyopathy, a pathophysiological concept of the abnormal atrial substrate and function, such as chamber dilation, impaired myocyte function, and fibrosis, is postulated to form a nidus for embolism. Several lines of evidence indicating the potential of atrial cardiomyopathy markers in the ESUS patients support this idea. From a practical viewpoint, anticoagulant therapy may be more beneficial than antiplatelet therapy in ESUS patients with atrial cardiomyopathy. There is an ongoing trial, Atrial Cardiomyopathy and Antithrombotic Drugs in Prevention After Cryptogenic Stroke (ARCADIA) trial, which is testing this hypothesis (4). Therefore, the proposed concept of atrial cardiomyopathy could stimulate us to better understand and identify the risk of ESUS, promoting the management of these patients in clinical practices.

## ESUS

### Definition and Prevalence

Even though CS accounts for one-third of IS, the definition of CS remains vague. TOAST criteria classify an IS as CS when no evidence can be identified even with sufficient evaluation of etiology. However, CS also consists of stroke with multiple causes and stroke with incomplete survey. Given the vague TOAST criteria and lack of agreement in the community, there has been a slow progress of the prevention in CS patients over the past decades. Most non-lacunar IS are embolic with a major clear source. However, there are many potential embolic sources, including subclinical atrial fibrillation (AF), atrial cardiomyopathy, patent foramen oval (PFO), cancer, non-stenotic artery atherosclerosis, nonatherosclerotic vasculopathies, and left ventricular disease. These causes may overlap in part and interact with each other.

Therefore, the term “embolic stroke of undetermined source” (ESUS) (3) was proposed in 2014 to describe non-lacunar IS patients without an identified cardioembolic source (including AF, valvular heart disease, intracardiac thrombus, cardiac tumors, and infective endocarditis), proximal arterial stenosis ≥ 50% (cervical or intracranial artery supplying the infarct area), and other determined uncommon stroke causes even after a standardized comprehensive evaluation. The term “ESUS” is a more specific concept to describe CS patients in whom embolic source is likely the underlying mechanism (3), which refines the category of CS, facilitating the progress of clinical trials to evaluate the potential of anticoagulant therapy to reduce the stroke recurrence in ESUS patients.

The diagnostic criteria (3) for ESUS include the following: (1) identification of non-lacunar IS by magnetic resonance imaging (MRI) or CT; (2) exclusion of ≥50% luminal stenosis in extracranial or intracranial arteries using MRI/CT-guided vascular imaging; (3) exclusion of major cardioembolic causes with ECG, echocardiography, and Holter monitoring; and (4) exclusion of other uncommonly determined causes of stroke (arteritis, dissection, migraine, and drug misuse). ESUS working group investigators further proposed that this clinic construct is a more clinically useful, definite concept than the vague term of CS (3). This construction uses more specified criteria to distinguish potential embolic sources from other clear sources.

Approximately 17% of IS patients fulfill the ESUS diagnostic criteria (ranging from 9 to 25%), who are typically younger patients (mean age of 65 years) with fewer systemic vascular risk factors, and severity of the strokes is often milder than other types of IS (defines NIHSS of 5) (5). However, the recurrence rate in ESUS averaged 4.5% per year, which is higher than that of non-ESUS IS (5). Given that the causes of ESUS remain unknown, nearly 86% ESUS patients were treated with antiplatelet therapy (5).

### Non-vitamin K Antagonist Oral Anticoagulants (NOACs) vs. Aspirin in ESUS

According to the hypothesis that many potential embolic sources result in ESUS, those patients may benefit from the anticoagulation treatment. There are two accomplished randomized trials to evaluate the efficacy of NOACs compared with aspirin in ESUS. The NAVIGATE ESUS trial (6) enrolled 7,213 participants to compare the efficacy and safety of rivaroxaban with aspirin for the prevention of ESUS patients. However, due to the high bleeding rate observed with rivaroxaban (hazard ratio = 2.72; 95% CI, 1.68–4.39; *P* < 0.001), this trial has been prematurely terminated. Disappointingly, the annual rate of primary outcomes (any recurrent stroke) did not significantly differ between the two groups (HR, 1.07; 95% CI, 0.87–1.33; *p* = 0.52). The RE-SPECT ESUS trial (7) compared the efficacy and safety of dabigatran with aspirin for the prevention of ESUS patients. There is a similar rate of first recurrent stroke (HR, 0.84; 95% CI, 0.68–1.03; *p* = 0.10) and similar safety outcomes (HR, 1.19; 95% CI, 0.85–1.66) in the two groups. In conclusion, although it was postulated that various underlying embolic sources contributing to ESUS would respond to anticoagulation favorably, unfortunately, neither NAVIGATE ESUS nor RE-SPECT ESUS found that oral anticoagulants were better than aspirin for secondary prevention in the ESUS patients.

In order to explore the value of anticoagulant therapy in secondary preventive treatment of ESUS patients, three randomized controlled trials have been conducted, and two of them have obtained results. However, it is disappointing that neither NAVIGATE ESUS trial nor RE-SPECT ESUS test can prove that anticoagulant therapy is more beneficial than antiplatelet therapy for ESUS patients, and the NAVIGATE ESUS trial is even terminated early because of the high incidence of severe bleeding events. The results of the ATTICUS ESUS trial using apixaban have not yet been published; considering that the inclusion criteria require ESUS patients have one of the risk factors of cardiac embolism, the results are highly anticipated.

The heterogeneity of mechanisms responsible for ESUS likely explains these unsatisfied results of the ESUS trials. There are various underlying embolic mechanisms in ESUS patients; the treatment strategy of ESUS patients should be formulated according to their own different embolic sources. By contrast, there is an overlap of underlying embolic sources in ESUS patients, more than 30% patients have ≥3 potential embolic sources, and each patient averagely had two underlying embolic sources (8). The benefit of anticoagulation was likely offset by other causes rarely benefitting from anticoagulation, such as those patients with non-stenosing large-artery atherosclerosis. Thrombi of atrial origin may need anticoagulants, such as subclinical AF, atrial cardiomyopathy, and patent foramen oval (PFO). On the contrary, arterial-origin thrombi in ESUS may be benefit from antiplatelet therapy for secondary prevention than anticoagulant therapy, including non-stenotic artery atherosclerosis, nonatherosclerotic vasculopathies, and left ventricular disease. Therefore, finding reliable diagnostic criteria to screen patients with different causes is necessary for individualized treatment, which may be the therapeutic target rather than the entirety concept of ESUS (9).

## Atrial Cardiomyopathy

### Definition and Prevalence

**Figure 2:**
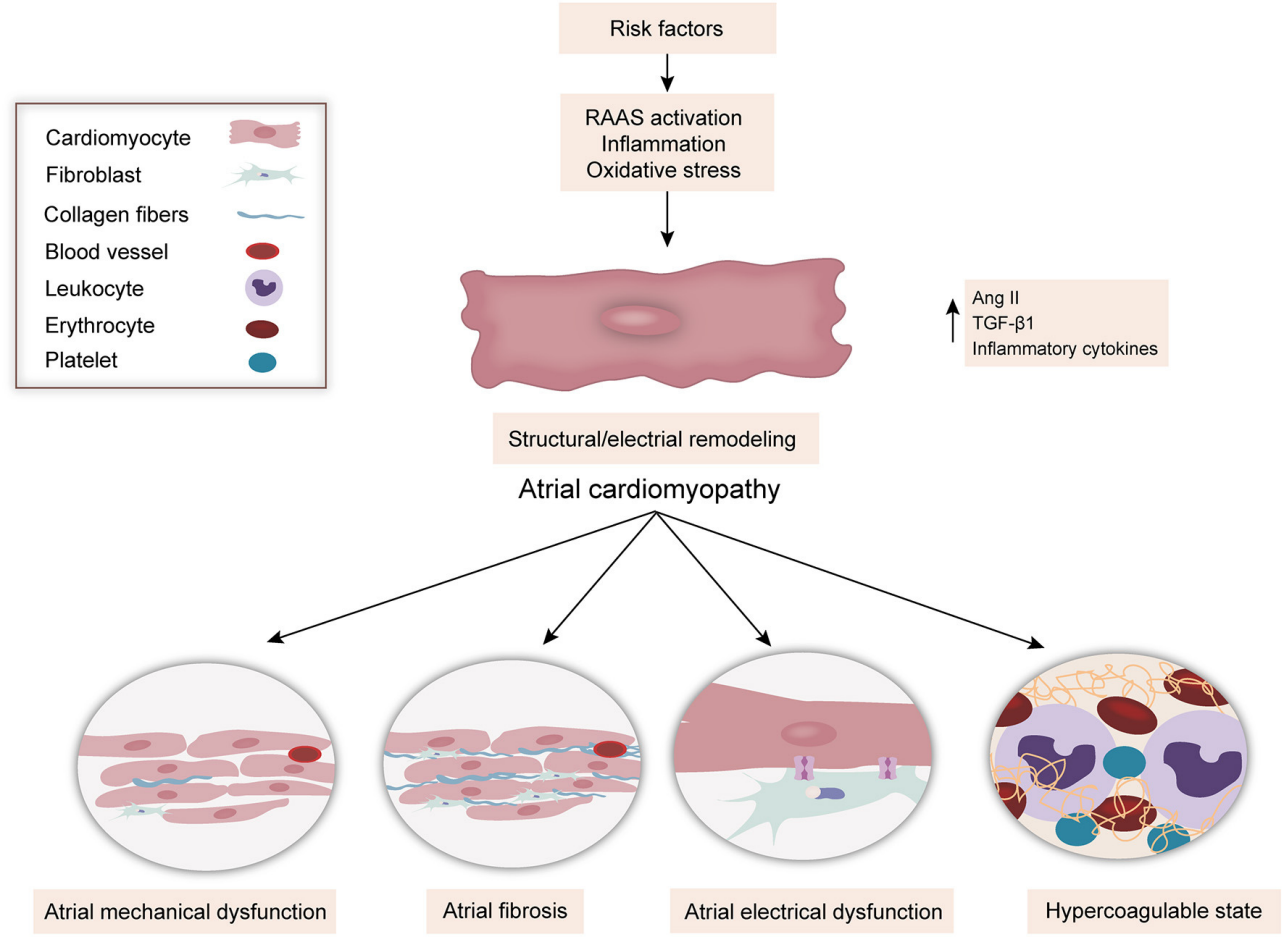
The classification of atrial cardiomyopathy. Multiple risk factors contribute to atrial injury which leads to atrial fibrosis, atrial mechanical dysfunction, atrial electrical dysfunction, and hypercoagulable state. Renin–angiotensin–aldosterone system, RAAS; Ang II, angiotensin II; TGF-β1, transforming growth factor-β1.

With the progress of relevant studies, atrial cardiomyopathy is considered as a common pathological feature of AF and an independent cause to the risk of stroke. The expert consensus defined atrial cardiomyopathy as “any complex of structural, architectural, contractile, or electrophysiological changes affecting the atria with the potential to produce clinically relevant manifestations” (10). Meanwhile, atrial cardiomyopathy was proposed (11) as a term to describe patients with abnormal atrial substrate and function, including atrial fibrosis, atrial mechanical dysfunction, atrial electrical dysfunction, and hypercoagulable state (Figure 2) (12), which can be present even without AF. The term “atrial cardiomyopathy” reframes our understanding of the association between AF and thromboembolism. Over the past decade, there have been many studies that focus on the underlying abnormal atrial structural and functional changes. AF and atrial cardiomyopathy have bidirectional interactions, with one predisposing to the other, and share common risk factors. This may explain why thromboembolism can be observed even in the absence of AF. Indeed, atrial cardiomyopathy may be the underlying cause of embolic stroke, similar to AF. Although there are no standard diagnostic criteria for atrial cardiomyopathy at present, different markers can be used to indicate atrial cardiomyopathy, for screening and evaluating stroke risk in ESUS patients. These include prolongation of the PR interval (13), abnormal P-wave terminal force in lead V1 (PTFV1) (14), prolonged P-wave durations (15), paroxysmal supraventricular tachycardia (PSVT) (16), left atrial enlargement (LAE) (17), and elevated cardiac biomarkers [e.g., N-terminal pro-brain natriuretic peptide (NT-proBNP) (18), cardiac troponin (cTnT) (18)].

Approximately 63% CS patients have an increased prevalence of markers of atrial cardiomyopathy (defined as NT-proBNP > 250 pg/mL, or PTFV1 > 5,000 μV·ms, or severe LAE) (19), and atrial cardiomyopathy (defined as LA > 38 mm for women and >40 mm for men or if supraventricular extrasystoles) was present in nearly 45% ESUS patients (8). Recent data showed that atrial cardiomyopathy, defined as PTFV1 > 5,000 μV·ms or severe LAE, occurred more frequently in ESUS patients than in another non-cardiogenic stroke (26.6 vs. 14.02%; *p* < 0.001) (20). All the studies are supportive of atrial cardiomyopathy as a possible embolic cause for ESUS.

### The Relationship Between Atrial Cardiomyopathy and AF

**Figure 3:**
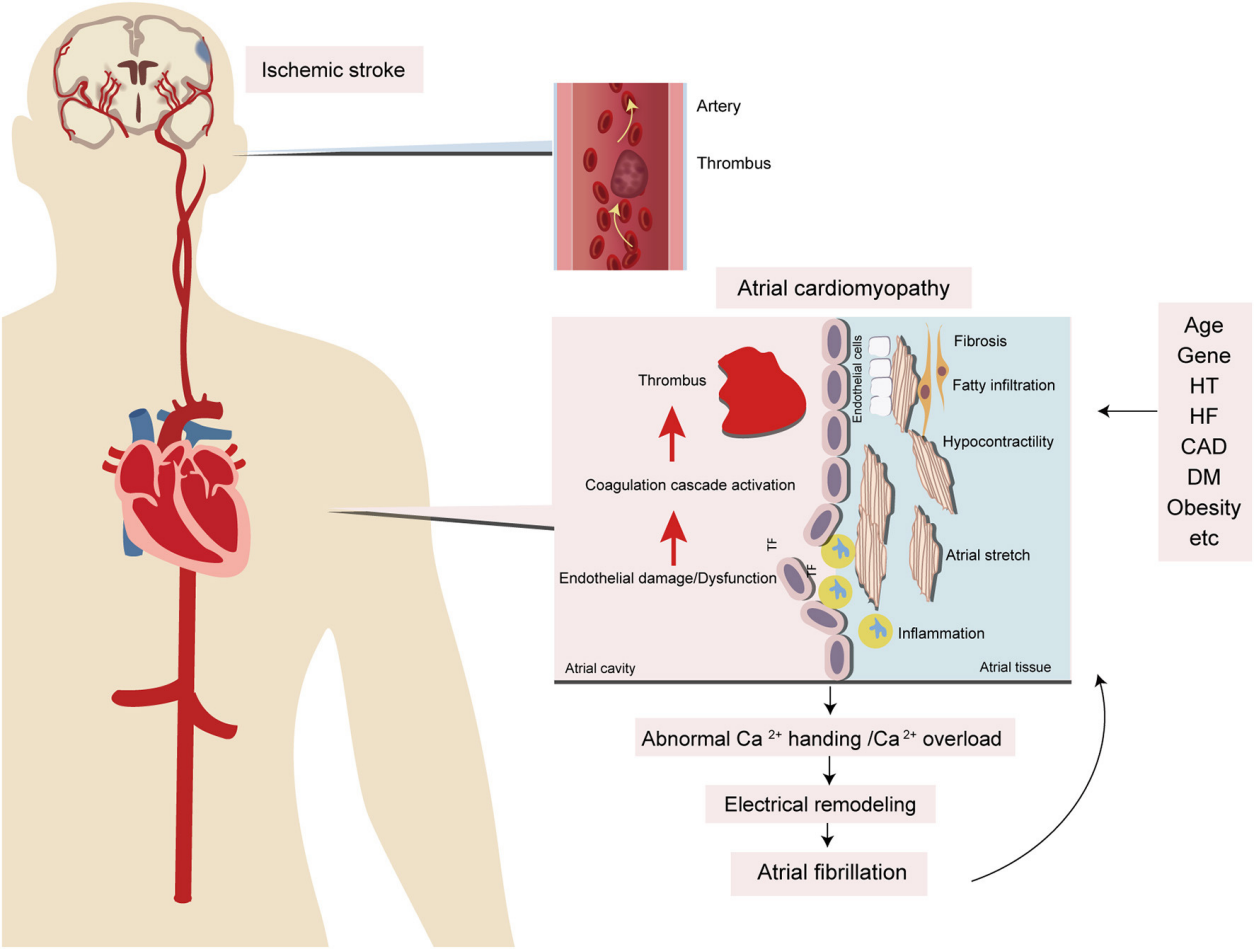
Potential mechanisms of atrial cardiomyopathy and the relationship with stroke and AF. Several risk factors, including aging, hypertension, heart failure, diabetes, obesity, inflammation, and obstructive sleep apnea, contribute to atrial injury, like stretch and enlargement which leads to atrial fibrosis, endothelial cell dysfunction, and impaired myocyte function. Because of these dysfunctions, there are electrical and structural remodelings of the myocardium that contribute to thromboembolism and AF. HT, hypertension; HF, heart failure; CAD, coronary artery disease; DM, diabetic mellitus; TF, tissue factor.

Given recent advances, atrial remodeling caused by several underlying cardiac diseases or systemic conditions is the fundament of the progression of AF. Conversely, AF itself can lead to atrial remodeling. Indeed, AF is a final common pathway of atrial remodeling caused by several cardiac or noncardiac conditions and AF itself can also contribute to atrial remodeling that leads to the progression of AF. Structural remodeling, including changes of atrial tissue, size, cellular ultrastructure, and especially fibrosis, has been believed to be the main cause of AF (21). Atrial fibrosis, the accumulation of fibrillar collagen deposits in the left atrial myocardium, manifests delayed-enhanced MRI (DE-MRI) that provides a noninvasive means to assess the myocardial tissue in AF patients, showing areas of fibrosis in the atria accurately (22, 23). Therefore, DE-MRI has been used to guide physicians to manage AF patients with catheter ablation. A recent study shows that left atrial fibrosis can also be detected by DE-MRI in a general cardiology population, even without structural heart disease or AF (24) and suggests that atrial fibrosis can present to the general population. DE-MRI plays an important role in the evaluation of potential cardiac causes. However, the relationship between atrial fibrosis on DE-MRI and stroke independent of AF remains unclear. Studies focusing on investigating this relationship are needed. Indeed, several risk factors can promote AF to occur, inducing change in atrial endocardial electrograms. The study found that advancing age results in greater abnormalities based on atrial endocardial electrograms recorded in patients without AF (25). By contrast, the imaging findings indicated that structural changes in the atria were significantly correlated with the presence and severity of AF (26, 27). The markers of atrial cardiomyopathy can predict the occurrence of AF (28, 29), but atrial cardiomyopathy does not recover even after successful catheter ablation for AF (30). However, management of vascular risk factors in AF patients after catheter ablation can effectively reduce the left atrial size by approximately half (31). Therefore, it is assumed that multiple cardiac and noncardiac conditions contribute to injury of the atrial substrate and cause atrial cardiomyopathy, which can result in AF and drive its progression; conversely, AF can worsen it through atrial remodeling (Figure 3).

### Clinical Significance

The relationship between AF and atrial cardiomyopathy reframes our understanding of embolic stroke. Given that AF is the consequence of atrial cardiomyopathy and the markers of atrial cardiomyopathy are strongly associated with stroke independently of AF, it is postulated that atrial cardiomyopathy can cause the embolic stroke even without AF. This may explain several questions about AF-related strokes. First, the ASSERT study (32) showed that only 8% individuals were detected with subclinical AF within the 30 days before stroke, and the TRENDS trial (33) reported that only 27.5% of patients were diagnosed with AF 30 days prior to the occurrence of cerebrovascular events or systemic emboli. These studies suggested that there is a temporal disassociation between AF and stroke. The CRYSTAL-AF investigation enrolled 441 CS patients to detect the prevalence of AF after CS used long-term monitoring with an insertable cardiac monitor (ICM) (34), suggesting that the result of detecting AF in the ICM group is more effective than the control group. Therefore, the more ambulatory electrocardiogram is done, the longer the duration, and the more likely AF will be detected, leading to improved treatment rates. However, given the limitation of cases in the subgroup, multiple studies are needed to evaluate the relationship between subclinical AF and stroke. Second, in a meta-analysis (35) of 28,836 patients, the rhythm-control therapy had no effect on stroke risk, suggesting that there are other underlying causes of stroke except dysrhythmia. Additional factors, such as atrial cardiomyopathy, may be the major contributors to stroke even without AF. Third, nearly 70% of patients with CS do not have detectable AF after 3 years of continuous heart-rhythm monitoring (34). Fourth, analysis of histological composition of the clots (36) showed that the clots of CS were similar to those of cardioembolic strokes, suggesting that the majority of CS have similar sources such as cardioembolic stroke. These lines of evidence indicate that AF is not a necessary factor in the progression of embolic stroke, and atrial cardiomyopathy may be the answer to these unsettled questions. From a practical viewpoint, it is reasonable to conclude that the concept of atrial cardiomyopathy can advance our ability to evaluate the stroke potential cardiogenic risks by markers of atrial cardiomyopathy without AF after prolonged heart-rhythm monitoring. In addition, recent work has shown that left ventricular wall motion abnormalities and changes in left heart function are potential sources of emboli in ESUS patients (37). Several studies also identified that accumulation of epicardial adipose tissue (EAT) around the left atrium is associated with increased risks of stroke (38). Future studies on the relationship between EAT around left atrium and different types of strokes are needed. As for the treatment and prevention, the secondary NAVIGATEESUS Trial analysis suggests that anticoagulant therapy may be more beneficial than antiplatelet therapy in ESUS patients with moderate or severe left atrial enlargement (39). Therefore, anticoagulant therapy could be more beneficial than antiplatelet therapy in ESUS patients with atrial cardiomyopathy.

### Mechanisms of Atrial Cardiomyopathy

AF frequently coexists with atrial abnormalities. These pathologically abnormal structures and functions can be induced by the interaction between various factors. These atrial abnormalities may lead to cardiomyocyte and interstitial remodeling, including electrical and structural changes that result in AF and thrombogenesis. By contrast, these atrial abnormalities, or atrial cardiomyopathy, have existed for some period before AF occurs (40). These evidences are supportive of the similar mechanisms underlying AF and atrial abnormalities (Figure 3).

Atrial cardiomyopathy not only occurs with aging but also results from many pathophysiological conditions, including systemic inflammatory conditions and low-grade subclinical inflammatory conditions (hypertension, heart failure, coronary artery disease, and so on) (41–43); these factors interact with each other, leading to activation of the renin–angiotensin–aldosterone system (RAAS) and production of angiotensin II (Ang II) with the potential to induce cardiomyocyte hypertrophy, endothelial abnormalities, and myocardial fibrosis. Ang II produces reactive oxygen species (ROS), leading to abnormal Ca^2+^ handling and Ca^2+^ overload, which contribute to electrical remodeling. Furthermore, Ang II produces transforming growth factor-β1 (TGF-β1), one of the key downstream efforts of Ang II that are secreted from cardiomyocytes and fibroblasts, which is a major factor in promoting fibrosis through the TGF-β1/Smad pathway to mediate the downstream gene product and connective tissue growth factor (CTGF), to increase atrial fibrosis and conduction abnormalities, and to promote AF. Eventually, these factors contribute to the electrical and structural remodeling of the myocardium. On the other hand, these inflammatory conditions result in inflammatory cells infiltrating the myocardium. Inflammatory cytokines promote the production of tissue factor (TF) and contribute to thrombogenesis. In addition, these inflammatory conditions can lead to endothelial injury, which promotes TF release from subendocardial tissue, further contributing to the coagulation cascade activation and leading to thrombogenesis (Figures 2, 3).

## Atrial Cardiomyopathy is the Contributory Factor of ESUS

In addition to subclinical AF, other factors may be involved in CS (34). Atrial cardiomyopathy was proposed to be associated with stroke independently of AF because ~65% CS patients have an increased prevalence of markers of atrial cardiomyopathy (11, 19). The results of the Cardiovascular Health Study (CHS) which evaluated the association between atrial cardiomyopathy markers and stroke risk showed that markers of atrial cardiomyopathy were each independently associated with incident stroke (44). Furthermore, atrial cardiomyopathy occurred more frequently in ESUS patients than in those other non-cardiogenic strokes, suggesting that atrial cardiomyopathy may be an underlying mechanism of ESUS (20).

Multiple vascular risk factors, such as aging, hypertension, diabetes, obesity, and obstructive sleep apnea, all of which are associated with a pro-inflammatory milieu, contribute to atrial dysfunction through stretch and enlargement, and eventually to atrial fibrosis, endothelial dysfunction, and impaired myocyte function. Because of these abnormalities, ineffective atrial contractile function can lead to blood stasis and formation of emboli in the atria or left atrial appendage (LAA), contributing to thromboembolism (Figure 3).

**Figure 4:**
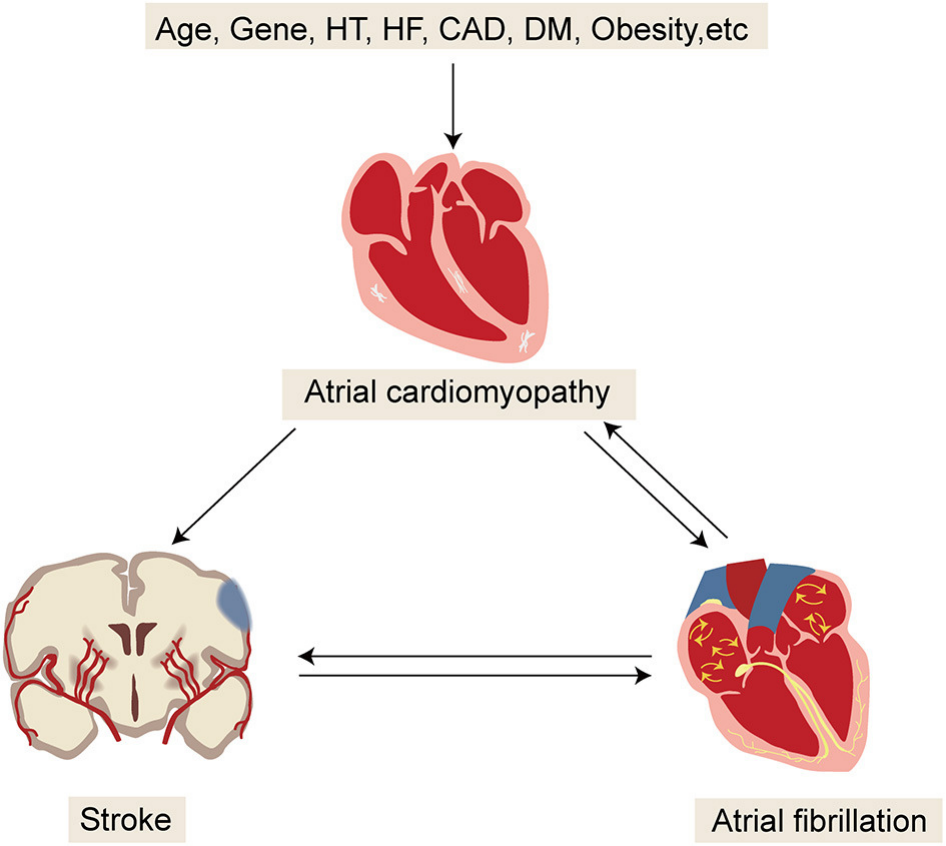
Updated model for better understanding the mechanisms of embolic stroke. HT, hypertension; HF, heart failure; CAD, coronary artery disease; DM, diabetic mellitus.

Recently, an updated model on the mechanisms of embolic stroke has been made available, which emphasizes the interacting factors among AF, atrial cardiomyopathy, and embolic stroke (Figure 4) (45). Multiple risk factors for stroke, such as aging and vascular risk factors, can undermine the atrial substrate, cause atrial cardiomyopathy, and subsequently increase the risk of AF and thromboembolism. By contrast, AF in turn leads to further worsening of atrial cardiomyopathy and thromboembolism. Once stroke occurs, autonomic changes and inflammation eventually increase the risk of AF. Conceivably, this model can help explain several puzzling observations about AF and stroke.

## Prospect

There has been increasing interest in recent years directed toward answering several open questions in the field of atrial cardiomyopathy and ESUS. The following unanswered questions merit further studies. First, the exact diagnostic criteria of atrial cardiomyopathy remain undecided, although some investigators hold the view that atrial cardiomyopathy can be diagnosed by the presence of one of the markers such as LAE, PTFV1, NT-proBNP, cTnT, and PSVT; the potential of each biomarker in diagnosing atrial cardiomyopathy remains unclear. By contrast, these markers are indirect in defining the atrial cardiomyopathy; future studies are needed to focus on the relationship between pathological features of atrial cardiomyopathy and ESUS. Second, although the markers of atrial cardiomyopathy are related to the risk of stroke occurrence independent of AF, the temporal relationship of atrial cardiomyopathy and IS remains unclear. Future studies need to investigate the sequence of disease onset. Third, to understand the atrial cardiomyopathy behind AF, we need to advance the ability to predict stroke and AF using a new scoring system. Fourth, the multicenter randomized controlled trials are necessary for comparing the potential of different therapies in patients with atrial cardiomyopathy without AF to reduce the risk of ESUS. The ongoing ARCADIA trial is testing this hypothesis (4).

In conclusion, the theoretical concept of ESUS is based on the fact that most CS is embolic; thus, anticoagulant therapy may be more beneficial for these patients. However, neither the NAVIGATE ESUS trial nor the RE-SPECT ESUS trial has confirmed this hypothesis, thinking that anticoagulant therapy would increase the risk of bleeding. However, considering the variety of potential embolic sources of ESUS, individualized treatments should be carried out according to each cause. Atrial cardiomyopathy may predispose patients to the high risk of ESUS. Several biomarkers can be used to sift patients with atrial cardiomyopathy. For ESUS patients with atrial cardiomyopathy, anticoagulant therapy may be more suitable. Further investigation of atrial cardiomyopathy as a modifiable stroke risk factor can help us better understand ESUS and choose a suitable option for ESUS patients, promoting the management of these patients in clinical practices.

## Author Contributions

YN and GLi drafted the manuscript, illustrated, and captioned all figures. YN, GT, GLi, and GLu provided the critical review, revision of the manuscript, and approved the final draft for publication. All authors contributed to the article and approved the submitted version.

## Conflict of Interest

The authors declare that the research was conducted in the absence of any commercial or financial relationships that could be construed as a potential conflict of interest.

## Publisher's Note

All claims expressed in this article are solely those of the authors and do not necessarily represent those of their affiliated organizations, or those of the publisher, the editors and the reviewers. Any product that may be evaluated in this article, or claim that may be made by its manufacturer, is not guaranteed or endorsed by the publisher.
